# The Extra Pharmacopœia

**Published:** 1890-06

**Authors:** 


*The Extra Pharmacopœia.* By WILLIAM MARTINDALE and W. WYNN WESTCOTT, M.B. Sixth Edition. London: H. K. LEWIS. 1890.

This book should be the companion of every medical man. With conciseness and in convenient form, it gives all that the prescriber needs to know of the preparations and therapeutics of the numerous drugs which, still on their trial, are not yet placed in the official list. The doses of all those that have reached that dignified position are given, and in certain instances some information is added about the preparations into which they enter. For the scientific enquirer the references to original investigations are numerous and clear. We were surprised to see urethane amongst the secondary list of drugs of which the authors have had little or no experience. The formulæ of the British Pharmaceutical Conference, 1889, are usefully included. Probably all these will appear in the next Addendum to the British Pharmacopœia, which will doubtless contain most of the drugs and preparations recommended by the Royal College of Physicians and by the committee of the General Medical Council.

All the wealth of information here provided is, by judicious arrangement, crowded into a volume which, notwithstanding successive editions are increasing in bulk, is still a small one; and although the doctor's pocket must be getting tolerably full, we strongly recommend him to get the book and keep it there.

